# Efficacy and safety of electroacupuncture for paralytic ileus in severe stroke: a protocol of multicenter, randomized controlled trial

**DOI:** 10.3389/fneur.2025.1615489

**Published:** 2025-10-24

**Authors:** Dehui Nie, Meiling Zhang, Bin Han, Dan Jin, Jianlong Huang, Liming Lu

**Affiliations:** ^1^Clinical Medical College of Acupuncture Moxibustion and Rehabilitation, Guangzhou University of Chinese Medicine, Guangzhou, China; ^2^Zhongshan Hospital of Traditional Chinese Medicine Affiliated to Guangzhou University of Chinese Medicine (Zhongshan Traditional Chinese Medicine Hospital), Zhongshan, China; ^3^Clinical Research and Big Data Laboratory, South China Research Center for Acupuncture and Moxibustion, Clinical Medical College of Acupuncture Moxibustion and Rehabilitation, Guangzhou University of Chinese Medicine, Guangzhou, China

**Keywords:** electroacupuncture, severe stroke, paralytic ileus, randomized controlled trial, gastrointestinal motility

## Abstract

**Introduction:**

Paralytic ileus is a frequent and serious complication of severe stroke, associated with poor prognosis and prolonged hospitalization. Current pharmacological options are limited by side effects and uncertain efficacy. Although electroacupuncture has shown promise in improving gastrointestinal motility, high-quality trials specifically targeting stroke-related paralytic ileus are still lacking, leaving an important evidence gap.

**Methods:**

This multicenter randomized controlled trial will recruit at least 100 severe stroke patients with paralytic ileus across China. Participants will be randomly assigned (1:1) to an electroacupuncture or sham electroacupuncture group. The electroacupuncture group will receive standardized electroacupuncture, while the sham electroacupuncture group will undergo sham acupuncture without electrical stimulation. The primary outcome is weekly spontaneous bowel movements. Secondary outcomes include bowel movement regularity, abdominal circumference, bowel sounds, emergency medication use, Glasgow Coma Scale, National Institutes of Health Stroke Scale, Modified Rankin Scale and length of hospitalization. Adverse events will be systematically monitored and recorded throughout the study. Efficacy will be assessed using linear mixed-effects models or generalized linear mixed models, with both intention-to-treat and per-protocol analyses performed.

**Discussion:**

The results of this multicenter RCT will provide robust evidence on the efficacy and safety of electroacupuncture for stroke-related paralytic ileus and may inform future clinical practice and guideline development.

**Clinical trial registration:**

https://itmctr.ccebtcm.org.cn/, identifier ITMCTR2025000003.

## 1 Introduction

Stroke ranks as the third leading cause of death and the fourth leading cause of disability-adjusted life-years (DALYs) worldwide (1). Among its complications, gastrointestinal dysfunction, particularly paralytic ileus, is a major concern in severe stroke cases (2, 3). Paralytic ileus, characterized by absent stool passage for three or more consecutive days without mechanical obstruction (4), affects over 40% of severe stroke patients (5, 6) and is commonly mistaken for constipation. In this vulnerable population, factors such as immobility, autonomic dysfunction, and medication side effects predispose to paralytic ileus (7). It contributes to abdominal distension, heightened risks of infection and malnutrition, and impairs neurorecovery by restricting rehabilitation and aggravating inflammation, placing considerable strain on patients and healthcare systems (8, 9).

Despite its clinical importance, paralytic ileus treatment remains conservative—nasogastric decompression, supportive care, and prokinetics (4, 5). Yet decompression poorly restores motility (10). Options like coffee or gum chewing aid recovery (11, 12) but face limitations in stroke patients: coffee may cause agitation; gum chewing is impractical in unconscious cases. Prokinetics, though common, show variable efficacy and risk of dependency or resistance (13–15). These limitations highlight the pressing need for safer and more efficacious therapeutic alternatives.

Electroacupuncture (EA), a type of acupoint stimulation therapy, has demonstrated potential in regulating autonomic function, enhancing gastrointestinal motility, and optimizing neurological outcomes (16, 17). Clinically, EA is proven to enhance postoperative bowel recovery, particularly in abdominal surgery cases, through improved gut motility (18, 19). However, current evidence for acupoint therapy in stroke ileus is weak, hampered by methodological issues such as inappropriate sham acupuncture controls or insufficient blinding (20, 21). Furthermore, severe stroke patients exhibit the highest incidence of paralytic ileus, yet EA’s therapeutic efficacy in this specific population remains largely unexplored.

Given these considerations, we have designed a randomized controlled trial (RCT) to assess the safety and efficacy of EA in alleviating paralytic ileus in patients with severe stroke.

## 2 Methods

### 2.1 Trial design and setting

**Figure 1:**
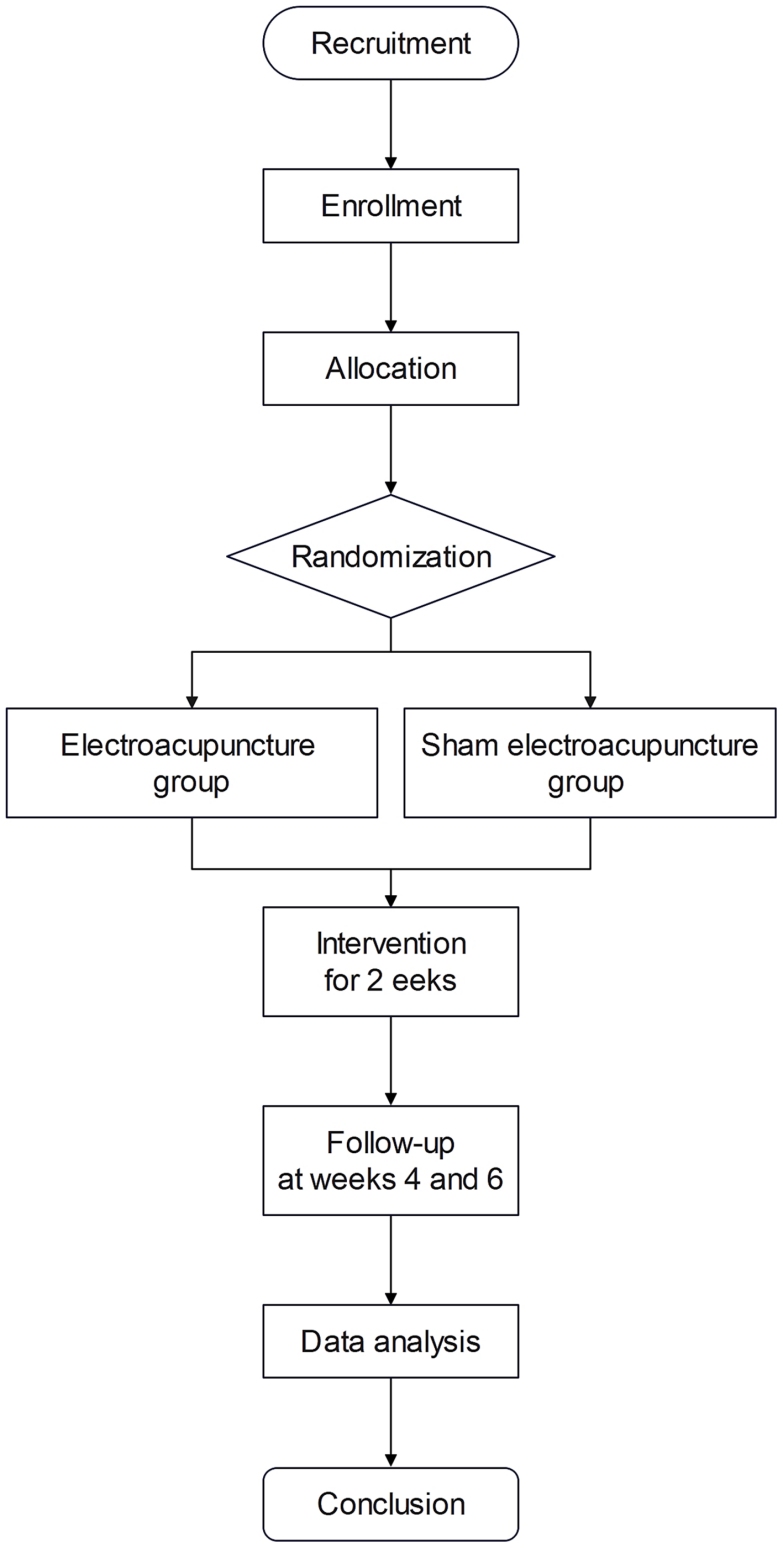
Study protocol flowchart.

This multicenter RCT will be conducted across three clinical centers in Guangdong, China. The study protocol follows the SPIRIT guidelines, and a detailed flowchart of the trial design is provided (Figure 1). The study protocol has been approved by the Medical Ethics Committees of all participating centers.

### 2.2 Patients

Participants will be recruited from the collaborating centers. Eligible patients will include those diagnosed with paralytic ileus (4) following severe stroke (22). Eligible patients will be screened per protocol.

#### 2.2.1 Inclusion criteria

1. Participants must meet the following criteria to be included in the study:
2. Diagnosis of severe stroke as defined by clinical assessment, with a Glasgow Coma Scale (GCS) score ≤ 12.
3. Clinically confirmed diagnosis of paralytic ileus, with the requirement of enteral nutrition for more than 48 h.
4. Age between 18 and 65 years, regardless of gender.
5. Stable vital signs for at least 24 h, with an Acute Physiology and Chronic Health Evaluation II score < 31.
6. For patients unable to provide informed consent, permission will be obtained from a direct family member before inclusion in the study.

#### 2.2.2 Exclusion criteria

1. Participants will be excluded from the study if they meet any of the following criteria:
2. Contraindications to acupuncture, such as severe dermatological conditions, bleeding disorders, or other contraindications to acupuncture therapy.
3. Severe dysfunction of the heart, liver, or kidneys, significant electrolyte disturbances, or other serious systemic diseases.
4. Pregnant or breastfeeding women.
5. History of severe primary gastrointestinal disorders, including inflammatory bowel disease, irritable bowel syndrome, gastrointestinal bleeding, gastrointestinal perforation, or functional constipation; or a history of major gastrointestinal surgery, such as stoma creation or extensive bowel resection.
6. Continuous agitation or status epilepticus, rendering the patient unable to cooperate with the study or potentially interfering with the study results.
7. Use of laxatives such as lactulose, or prokinetic agents such as neostigmine, prior to enrollment in the trial.

### 2.3 Randomization and blinding

Eligible participants will be randomly and equally assigned to the EA group or the sham electroacupuncture (SEA) group using a central web-based randomization system (http://47.119.117.255). The randomization sequence will be generated with a block size of 4. An independent researcher, who is not involved in outcome assessment, will perform the randomization and group allocation.

Since acupuncture involves manual procedures, only the acupuncturists will be unblinded. Outcome assessors, physicians, nurses, caregivers, patients, and statisticians will all remain blinded. An independent monitor will be responsible for maintaining the integrity of the blinding process.

### 2.4 Intervention

**Figure 2:**
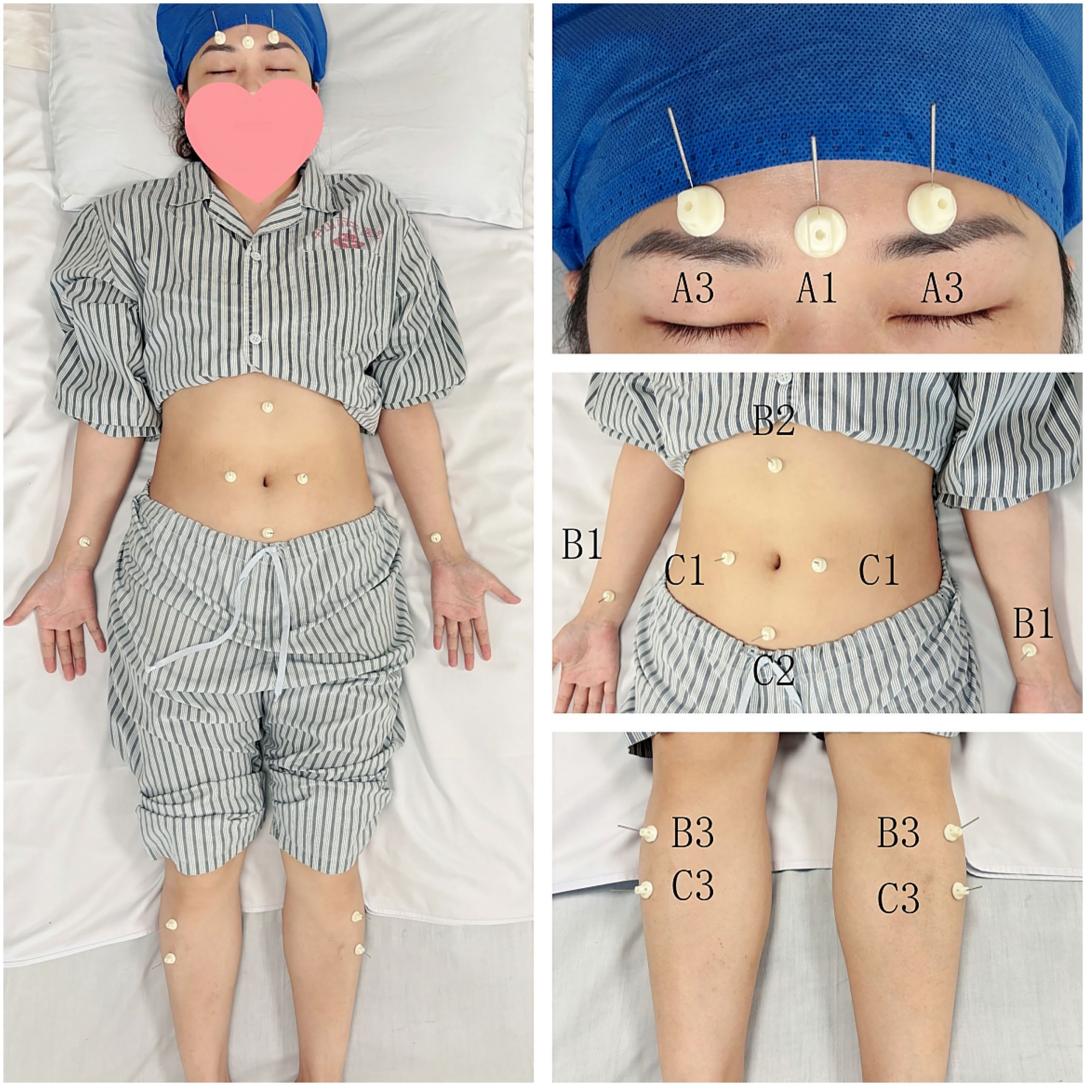
Acupoint anatomical locations. A1, Dingshen I; A2, Dingshen II; A3, Dingshen III; B1, Neiguan (PC6); B2, Zhongwan (CV12); B3, Zusanli (ST36); C1, Tianshu (ST25); C2, Guanyuan (CV4); C3, Shangjuxu (ST37).

Licensed acupuncturists with at least 3 years of experience will administer the interventions. The protocol, grounded in traditional Chinese acupuncture theory and refined through expert consultations, employs Jin’s Three-Needle Therapy. The “Dingshen-zhen,” “Weisan-zhen,” and “Changsan-zhen” acupoint groups are targeted for their effectiveness in gastrointestinal and neurological conditions (Figure 2). See Supplementary material for more details.

**Figure 3:**
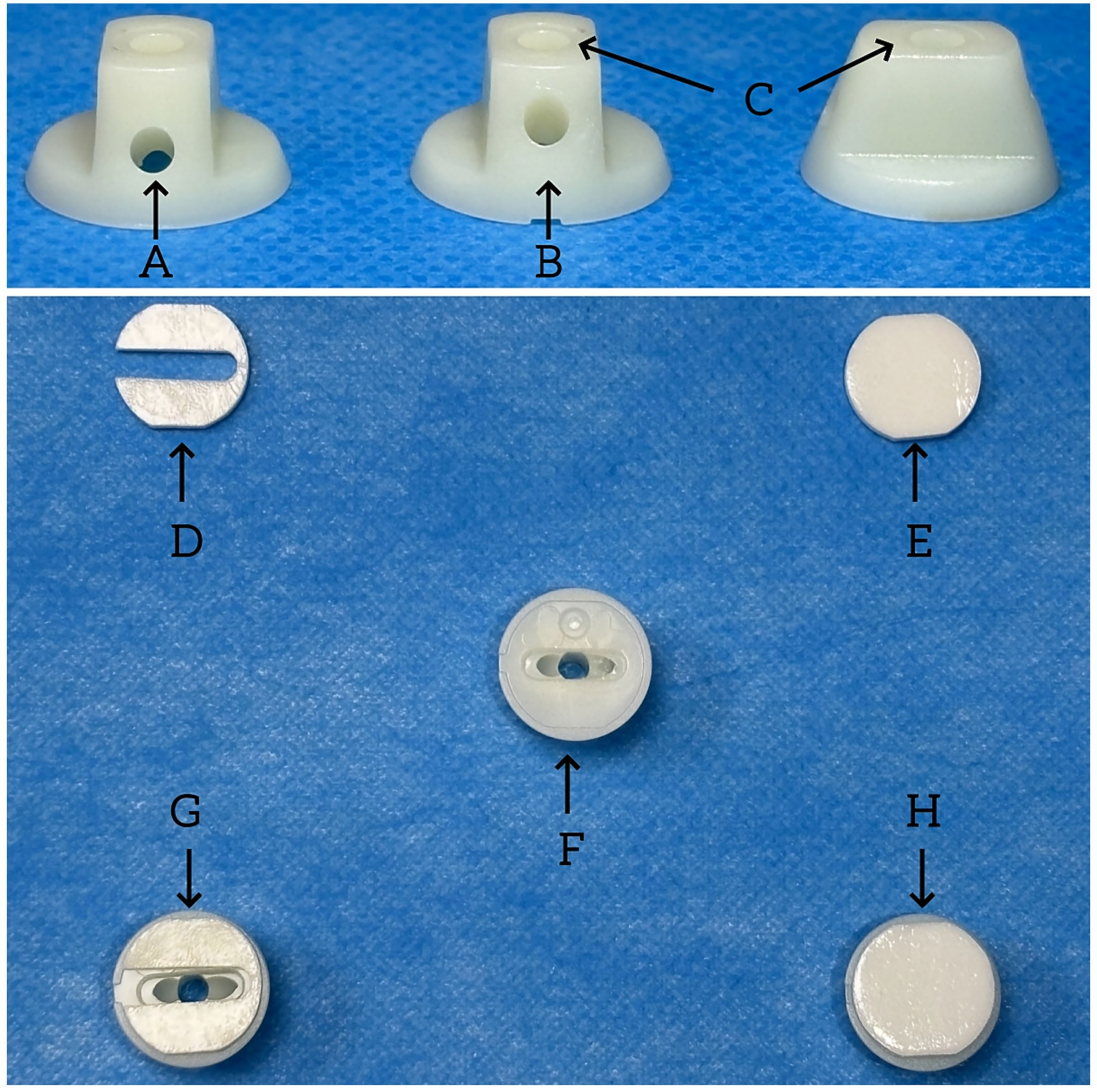
Patient-blinded acupuncture auxiliary device. **(A)** horizontal insertion; **(B)** oblique insertion; **(C)** vertical insertion; **(D)** hollow adhesive patch; **(E)** non-hollow adhesive patch; **(F)** acupuncture auxiliary device; **(G)** hollow instruments for electroacupuncture; **(H)** non-hollow instruments for sham electroacupuncture.

To ensure patient blinding, both groups will utilize a Patient-Blinded Acupuncture Auxiliary Device, previously validated in our studies for its effectiveness in maintaining blinding integrity (23). The device includes a plastic pedestal and three guide tubes, facilitating vertical, oblique, and horizontal needle insertions (Figure 3). Its design is identical for both groups.

#### 2.4.1 EA group

In the EA group, hollow instruments are used to allow sterile needle insertion. Sterile, disposable stainless steel acupuncture needles (Huatuo; 25 × 0.3 mm and 40 × 0.3 mm) with sharp tips will be inserted into predefined acupoints to an appropriate depth. Manual manipulation will be performed to elicit the deqi sensation. Electroacupuncture will then be administered using the Hua Tuo SDZ-II device. Electrodes will be connected from the right ST25 to CV12 and from the left ST25 to CV4, with continuous stimulation at 10 Hz and 0.5–4 mA for 30 min.

#### 2.4.2 SEA Group

Participants in the SEA group will receive an identical-appearing intervention to ensure effective blinding. The same patient-blinded auxiliary device will be applied, using non-hollow instruments and blunt-tip needles that do not penetrate the skin. A pricking sensation will be simulated to mimic the tactile experience of real acupuncture.

Electrodes will be placed at the same acupoints as in the EA group. However, no electrical current will be delivered. The procedure is designed to maintain visual and procedural consistency with the EA group while avoiding actual needle insertion or stimulation.

#### 2.4.3 Basic treatment

Basic supportive care includes continuous monitoring of vital signs to enable early detection of clinical deterioration. Oxygen therapy is provided as needed to prevent or correct hypoxemia and ensure adequate tissue oxygenation. For patients requiring mechanical ventilation, individualized ventilatory strategies are implemented and regularly adjusted based on respiratory parameters and clinical response. Fluid and electrolyte balance is actively maintained throughout the hospitalization, with adjustments guided by laboratory findings and the patient’s clinical condition. Progressive enteral nutrition support is provided in Supplementary material, along with infection management, glycemic control, and other supportive treatments as clinically indicated.

#### 2.4.4 Rescue medication

If a patient has no bowel movement for three or more consecutive days, a glycerin suppository (Glycerin Enema) will be administered rectally to stimulate evacuation (24). If constipation persists for another 3 days after enrollment or during treatment, bowel-regulating medication should be initiated promptly, with dosage and frequency recorded. Bowel movements within 24 h after suppository administration will not be counted as spontaneous.

#### 2.4.5 Follow-up

Patients will have video or in-person follow-ups at Weeks 4 (2 weeks after treatment) and 6 (4 weeks after treatment).

### 2.5 Data management

Study data will be recorded on case report forms (CRFs) by assessors, with participants identified by randomization numbers. Two independent staff will perform double data entry. The data management system will validate entries for range and logic errors. All data will be encrypted, with access restricted to authorized personnel. Paper CRFs will be securely stored at the South China Research Center for Acupuncture and Moxibustion for no less than three years after the completion of the study.

### 2.6 Quality control

The trial will use web-based randomization and standardized training to minimize bias. All data will be entered in real-time via CRFs, with three-tier monitoring of outcomes, documentation, and data management.

### 2.7 Sample size

This RCT evaluates Weekly Spontaneous Bowel Movements (WSBM) as the primary outcome, aiming to compare the mean differences between the two groups. The sample size was estimated using PASS 15 software, based on preliminary data from a pilot study (WSBM: 7.11 ± 2.20 in the EA group and 5.71 ± 1.50 in the SEA group). Given a significance level (*α*) of 0.05 and a power (1-*β*) of 0.9, the required sample size per group was determined to be 40 participants. Considering a 20% dropout rate, at least 100 participants will be enrolled. Additional recruitment may be conducted if feasible.

### 2.8 Statistical analysis

This study will employ both intention-to-treat analysis and per-protocol analysis. The intention-to-treat analysis will include all randomized participants, while the per-protocol analysis will focus on those with good compliance and no major protocol violations. The safety analysis set will comprise participants who receive at least one intervention and have safety evaluations.

All statistical analyses will be conducted using SAS software (version 9.4; SAS Institute Inc., Cary, NC, USA). Two-sided tests will be used, with *p* < 0.05 considered statistically significant. Continuous variables will be assessed for distribution using histograms and Q–Q plots, and reported as mean ± standard deviation (SD) or median (interquartile range, IQR), as appropriate. Categorical variables will be presented as counts (n) and percentages (%). Between-group comparisons will use independent t-tests or Mann–Whitney U tests for continuous variables, and Chi-square or Fisher’s exact tests for categorical variables.

The primary outcome, WSBM, is a discrete quantitative variable assessed at multiple time points. Depending on the data distribution, either a linear mixed-effects model or a generalized linear mixed model will be used. Secondary outcomes with continuous repeated measures, such as bowel sound frequency, GCS, and the National Institutes of Health Stroke Scale (NIHSS), will be analyzed using linear mixed-effects models if the data are approximately normally distributed. Otherwise, generalized linear mixed models with appropriate distributions will be applied. Rescue medication use, as a count variable assessed over time, will be analyzed using generalized linear mixed models. The Modified Rankin Scale (mRS), being an ordinal variable assessed repeatedly, will be analyzed using ordinal logistic regression models for repeated measures. Length of hospital stay, a continuous variable, will be compared between groups using either the independent samples t-test or the Mann–Whitney U test, depending on distribution. Adverse events, as categorical variables, will be analyzed using the chi-square test or Fisher’s exact test, depending on the expected cell counts. The primary outcome will be analyzed as the sole confirmatory endpoint, while all secondary outcomes will be treated as exploratory. For repeated measures, *post hoc* correction will be applied, but no formal multiplicity adjustment will be conducted across multiple secondary endpoints. Additional statistical analyses are provided in the Supplementary material.

## 3 Outcomes

### 3.1 Primary outcomes

WSBM is assessed by recording the number of naturally occurring bowel movements within a week (25). This indicator was selected as the primary outcome due to the high incidence and recurrence of paralytic ileus in patients with severe stroke. Scores range from 0 upward, with higher scores indicating better recovery of intestinal function (26).

### 3.2 Secondary outcomes

Assessment of Bowel Movement Regularity involves daily monitoring of spontaneous bowel movements over a week. A score of 1 is assigned for each day with a spontaneous bowel movement occurring without the use of emergency medication and without diarrhea, while a score of 0 is given if no bowel movement occurs, if emergency medication is used, or if diarrhea is present. The total score ranges from 0 to 7, with higher scores indicating better bowel movement regularity and overall intestinal function.

Rescue medication will be carefully recorded throughout both the treatment and follow-up periods, with detailed documentation of the frequency, dosage, and timing of administration. Increased use of rescue medication will be considered a potential indicator of the severity and recurrence rate of gastrointestinal dysmotility.

Bowel sounds are assessed via auscultation with the patient in a supine position. Each abdominal quadrant (right upper, right lower, left lower, and left upper) is auscultated for 1 min, and the average frequency is calculated. Within a physiologic range, higher average bowel sound frequencies indicate better intestinal motility and recovery (4).

Abdominal circumference is measured to assess distension and gastrointestinal function recovery. With the patient supine and relaxed, a measuring tape is placed horizontally at the umbilicus level, snug but not compressing the skin. The measurement, recorded in centimeters (cm), is taken at the end of gentle exhalation, repeated twice, and averaged. Higher values indicate severe distension and impaired function, whereas lower values suggest improvement. A decrease over time is considered favorable (27).

GCS is used to evaluate neurological status, monitor changes in consciousness, and predict prognosis. It assesses consciousness through three components: eye response, verbal response, and motor response (28). The total score ranges from 3 to 15, with higher scores indicating better consciousness and neurological function.

NIHSS is used to evaluate the severity of neurological deficits in stroke patients. It consists of 15 items assessing consciousness level, language ability, visual fields, motor function, sensory function, ataxia, and coordination (29). Scores range from 0 to 42, with higher scores indicating more severe neurological impairment and greater stroke severity.

mRS is used to evaluate long-term functional outcomes and quality of life after stroke (29). It assesses disability and dependence, ranging from 0 (no symptoms) to 6 (death), with higher scores indicating greater functional impairment.

Length of hospitalization, measured from admission to discharge, reflects overall recovery. Shorter stays indicate better outcomes, while prolonged stays suggest delayed recovery and greater dysfunction. Further details of standardized outcome assessment procedures are provided in Supplementary material.

Study outcomes are summarized in Table 1.

**Table 1:** Overview of study outcomes.

Outcome	Data range	Interpretation
Primary outcomes		
Weekly spontaneous bowel movements	≥0 (times/week).	Higher values indicate better intestinal function.
Secondary outcomes		
Bowel movement regularity	0–7 (score/week).	Higher scores indicate better regularity.
Rescue medication use	-	More use indicates worse symptoms.
Bowel sounds	≥0 (times/min).	Higher frequency indicates better motility.
Abdominal circumference	-	Lower values suggest improvement over time.
Glasgow coma scale	3–15 score.	Higher scores indicate better consciousness.
National institutes of health stroke scale	0 - 42score.	Higher scores indicate more severe impairment.
Modified rankin scale	0–6 score.	Higher scores indicate greater disability.
Length of hospitalization	3–45.	Shorter stay indicates faster recovery.

### 3.3 Safety evaluation

All adverse events will be recorded on paper CRFs by the acupuncturist, including the time of occurrence, severity, management, and their relationship to the intervention. Mild adverse events will include minor bleeding, bruising, acupuncture-related pain, and localized infection; serious events will include organ injury and uncontrolled hemorrhage. In the event of serious adverse events, the intervention will be stopped immediately, appropriate treatment will be provided, and the event will be reported to the Institutional Review Board within 24 h.

The timeline for each assessment is detailed in Table 2.

**Table 2:** Timeline of each assessment.

Outcomes	Intervention			Follow-up	
	Week 0	Week 1	Week 2	Week 4	Week 6
Weekly spontaneous bowel movements		**×**	**×**	**×**	**×**
Bowel movement regularity		**×**	**×**	**×**	**×**
Rescue medication use		**×**	**×**	**×**	**×**
Bowel sounds	**×**	**×**	**×**		
Abdominal circumference	**×**	**×**	**×**		
Glasgow coma scale	**×**	**×**	**×**	**×**	**×**
National institutes of health stroke scale	**×**	**×**	**×**	**×**	**×**
Modified rankin scale	**×**	**×**	**×**	**×**	**×**
Length of hospitalization					×
Safety evaluation	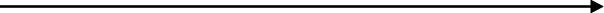				

## 4 Discussion

This study represents the first RCT specifically designed to evaluate the efficacy of EA in treating severe stroke-related paralytic ileus. While EA has been explored in postoperative ileus (18, 19), its application in critically ill stroke patients remains under-investigated.

The therapeutic effects of EA on paralytic ileus are hypothesized to involve multiple potential pathways. First, EA may modulate autonomic nervous function, particularly by enhancing vagal tone (30). Second, EA could regulate gastrointestinal hormones, such as motilin and gastrin, thereby improving intestinal peristalsis (31). Third, EA may exert anti-inflammatory effects by reducing pro-inflammatory cytokines, thus alleviating gut dysmotility (32). Additionally, emerging evidence suggests EA may alter gut microbiota composition (33), potentially restoring a favorable intestinal microenvironment conducive to motility recovery.

Compared with previous studies (18, 19) that predominantly focused on postoperative ileus and relied heavily on single-endpoint measurements such as time to first flatus or defecation, the present trial provides two significant methodological advancements. First, we selected WSBM as the primary outcome, which offers a continuous and objective measurement of sustained bowel function recovery, especially advantageous in stroke patients who cannot accurately report symptoms (7, 34). Unlike time to first flatus or defecation, it captures sustained improvements, offering a more meaningful efficacy assessment. Second, we employed a rigorous sham-controlled design using placebo needles, enhancing blinding effectiveness and reducing biases arising from patient expectations, thereby improving the internal validity and reliability of the findings (23).

Clinically, the findings from this trial could substantially influence practice by validating EA as an effective and safe therapeutic option for managing gastrointestinal dysfunction in critically ill stroke patients. If effective, EA may become an integral part of multimodal treatment strategies in intensive care units, thereby enhancing patient recovery, reducing the use of pharmacological laxatives, and improving overall quality of care. Moreover, positive results could support the wider clinical adoption of acupuncture-based interventions, potentially reducing complications related to impaired gut motility, shortening hospitalization, and informing future guideline recommendations for the management of stroke-related paralytic ileus.

This multicenter trial may face challenges in ensuring the consistency of acupuncture procedures across different study sites. First, all acupuncturists will undergo standardized training and obtain certification prior to patient enrollment, with continuous quality control measures implemented throughout the study to ensure procedural fidelity. Although no formal blinding index will be conducted, the use of a validated patient-blinded auxiliary device, together with centralized monitoring, is expected to maintain the integrity of blinding and minimize inter-centre variability. Second, potential adherence issues, particularly in the critical care context, will be addressed through enhanced communication strategies, structured education for healthcare providers, and routine follow-up with patients and their families. Third, for patients with impaired consciousness, a multidimensional outcome assessment system will be adopted to ensure objectivity and reliability in evaluations. Finally, all intercurrent events and instances of missing data will be systematically recorded. Sensitivity and subgroup analyses will be conducted to assess their potential impact on the study outcomes and to ensure the robustness of the results.

In conclusion, this rigorously designed RCT is expected to provide robust evidence for the efficacy and safety of EA in severe stroke-related paralytic ileus, while also offering important translational insights that may inform clinical decision-making and future guideline development.
