# Supplementary material for: Efficacy and safety of electroacupuncture for paralytic ileus in severe stroke: a protocol of multicenter, randomized controlled trial
Source: Front Neurol. 2025 Oct 24;16:1615489. doi: 10.3389/fneur.2025.1615489 (PMC12593516; doi:10.3389/fneur.2025.1615489)
Supplement: Supplementary file 1 [file Data_Sheet_1.pdf]

Supplementary Material

1 Intervention

1.1 The Rationale for Acupoint Selection

The acupoint selection protocol for this study was developed through a thorough integration of theoretical principles and clinical practice, refined via multiple rounds of expert consultations and preliminary trials. During the protocol development phase, the research team convened several preparatory meetings, inviting senior experts in acupuncture and neurocritical care to engage in in-depth discussions on the pathophysiology of intestinal paralysis in severe stroke patients, clinical experiences with acupuncture interventions, and evidence from existing literature. By systematically synthesizing expert opinions, the team formulated a standardized acupuncture treatment protocol centered on Jin's Three-Needle Therapy (JTN).

This protocol not only adheres to traditional Chinese medicine theories but also incorporates insights from modern research. Preliminary small-scale trials demonstrated its favorable safety profile and clinical efficacy, ultimately leading to the final standardization of the acupuncture intervention in this study.

The selected acupoints are derived from Jin' s Three-Needle Therapy (JTN), a clinically established acupuncture system that has been successfully applied in several randomized controlled trials. JTN utilizes a synergistic combination of three acupoints as a functional group to enhance therapeutic outcomes. In this study, the Dingshen-zhen (定神针), Weisan-zhen (胃三针), and Changsan-zhen (肠三针) acupoint groups were chosen to optimize treatment efficacy.

1.2 Acupoints and Locations

"Dingshen-zhen": Dingshen I, Dingshen II, Dingshen III

"Weisan-zhen": Neiguan, Zhongwan, Zusanli

"Changsan-zhen": Tianshu, Guanyuan, Shangjuxu

See Table 1 for details.

Table 1. Acupoint Locations

Acupoints	Code	Description
Dingshen I	NA	Located 0.5 cun above Yintang (EX-HN3)
Dingshen II	NA	Located 0.5 cun above left Yangbai (GB14)
Dingshen III	NA	Located 0.5 cun above right Yangbai (GB14)
Neiguan	PC6	Located on the anterior side of the forearm, 2 cun above the transverse crease of the wrist, between the tendons of the

		palmaris longus and the flexor carpi radialis.
Zhongwan	CV12	Located on the upper abdomen, 4 cun above the umbilicus, on the anterior midline.
Zusanli	ST36	Located on the lateral side of the lower leg, 3 cun below Dubi (ST35), on the line connecting Dubi (ST35) and Jiexi (ST41).
Tianshu	ST25	Located on the upper abdomen, at the level of the umbilicus, 2 cun lateral to the anterior midline.
Guanyuan	CV4	Located on the lower abdomen, 3 cun below the umbilicus, on the anterior midline.
Shangjuxu	ST37	Located on the lateral side of the lower leg, 6 cun below Dubi (ST35), on the line connecting Dubi (ST35) and Jiexi (ST41).

1.3 Operational Details of Intervention

EG will utilize disposable sterile stainless steel needles. Each needle will be inserted to a specified depth at the acupoints (see Table 2 for details). To enhance the "Deqi" sensation, the needles will be manipulated using insertion, twisting, and lifting techniques. For electroacupuncture, the right Tianshu (ST25, negative electrode) will be connected to Zhongwan (CV12, positive electrode), and the left Tianshu (ST25, negative electrode) will be connected to Guanyuan (CV4, positive electrode) using crocodile clips attached to the electroacupuncture device (Hua Tuo SDZ-II). A continuous wave at 10 Hz and an intensity of 0.5–4 mA will be applied, adjusted to elicit visible muscle twitching.

The sham electroacupuncture group will receive treatment at the same acupoints as the electroacupuncture group. Disposable blunt-end needles will be used to apply mild pressure at each acupoint without skin penetration or elicitation of the "Deqi" sensation. The right Tianshu (ST25) will be connected to Zhongwan (CV12), and the left Tianshu (ST25) will be connected to Guanyuan (CV4) using crocodile clips attached to the electroacupuncture device (Hua Tuo SDZ-II); however, no electrical stimulation will be applied.

Both groups use identical acupoints, but the electroacupuncture group applies sharp needles with electrical stimulation to induce "Deqi," while the sham electroacupuncture group uses blunt needles without skin penetration or electrical stimulation.

Table 2. Technical Details of Intervention

Acupoints	Direction	Depth		Electrode
		EA (mm)	SEA(mm)	
Dingshen I	H	15-25	0	N/A
Dingshen II	H	15-25	0	N/A
Dingshen III	H	15-25	0	N/A
Neiguan	V	15-25	0	N/A
Zhongwan	V	25-40	0	Positive
Zusanli	V	25-40	0	N/A
Tianshu (L)	V	25-40	0	Negative
Tianshu(R)	V	25-40	0	Negative
Guanyuan	V	25-40	0	Positive
Shangjuxu	V	25-40	0	N/A

Note: EA, electroacupuncture group; SEA, sham electroacupuncture group; H, horizontal insertion; V, vertical insertion.

1.4 Patient-Blinded Acupuncture Auxiliary Device

The intervention employed a Patient-Blinded Acupuncture Auxiliary Device (Patent No. ZL 202223328917.3, China). The device includes a plastic pedestal and three guide tubes, facilitating vertical, oblique, and horizontal needle insertions. Its design is identical for both groups. For EA, hollow instruments enable sterile needle insertion, while SEA uses non-hollow instruments with blunt needles. To maintain single-blind conditions, the acupuncturist secures the auxiliary device and needle (real or sham) to the sterilized acupoint. A quick tap on the tube enables the needle to either penetrate the skin (real) or remain on its surface (sham), and the tube is then removed. This standardized process ensures participant blinding to group allocation and intervention type.

2 Basic treatment

2.1 Monitoring & Vital Support

Continuous monitoring of vital signs (BP, HR, RR, SpO_2) ensures early detection of abnormalities. Oxygen therapy will be provided as needed via nasal cannula or face mask. For mechanically ventilated patients, ventilatory parameters (modes, tidal volumes, RR) will be adjusted accordingly. Fluid and electrolyte balance will be carefully maintained.

2.2 Nutritional Support & Assessment

Given the high prevalence of consciousness impairment/dysphagia in stroke patients, enteral nutrition will be administered via feeding pump over 12–24 hours, targeting 25–30 kcal/kg/day and 1.2–2.0 g/kg/day of protein. During the first 72 hours, caloric intake will be limited to <70% of the target, increasing to 70–100% within the first ICU week. Specialized enteral formulas will be used for diabetic patients when needed. Across centers, commercially available standard polymeric formulas (e.g., Ensure®, Resource®) will be used as the first choice, while specialized formulas (e.g., Glucerna®) will be applied for diabetic patients when clinically indicated.

3 Outcome assessment standardization

3.1 Patient/caregiver Education

Upon enrollment, family members and primary caregivers will receive structured education provided by trained study staff. The education will include detailed instructions on how to record bowel movements, including the frequency (number of spontaneous bowel movements per day and per week), the approximate amount of stool, and the use of rescue medications, including the medication name, dosage, time of administration, and reason for use. Caregivers will receive standardized recording forms or guidance on using recommended mobile applications for real-time documentation. To ensure proper understanding, training will be conducted through face-to-face or video sessions, incorporating demonstrations and practice exercises. Caregivers will be asked to complete sample records for review by the research team to confirm their proficiency. During the study, the research team will regularly review submitted records, provide timely feedback, and offer additional support through phone calls or messages as needed. Caregivers will be encouraged to maintain continuous recording until the end

of the follow-up period. This structured educational process will help ensure the accuracy and reliability of data collected for outcome assessment.

3.2 Research staff training

All investigators involved in outcome assessment received standardized training before the start of the trial, covering procedures such as bowel sound auscultation, abdominal circumference measurement, bowel movement regularity recording, and neurological scale evaluation. Only those who passed the training assessment were qualified to perform evaluations. Each center designated two fixed research staff members to carry out the assessments, with a third person involved for verification when necessary to minimize inter-rater variability and ensure data accuracy and consistency.

In this study, bowel sounds and abdominal circumference were measured for each patient at relatively fixed time periods, ensuring that the same patient was assessed at consistent times before treatment and at subsequent follow-up points, thereby reducing the influence of circadian variation on gastrointestinal function. For bowel sound assessment, the patient was placed in a supine and quiet state, and auscultation was performed sequentially in the right upper, right lower, left lower, and left upper abdominal quadrants for one minute each. The bowel sound frequency was recorded and the average calculated, with higher average frequencies within the physiological range indicating better intestinal motility and recovery. Abdominal circumference was then measured with the patient still supine and relaxed. A measuring tape was placed horizontally at the level of the umbilicus, snug but without compressing the skin, and the measurement was taken at the end of gentle exhalation. Two consecutive measurements were recorded and averaged. To minimize measurement error from the tools, all centers used measuring tapes of the same brand and batch, with regular replacement to avoid stretching-related bias. Neurological scales (such as GCS, NIHSS, and mRS) were also assessed independently and simultaneously by the two research staff members. In cases where discrepancies occurred, results were discussed until consensus was reached, thereby improving the reliability and reproducibility of the evaluations.

4 Statistical analysis

4.1 Demographic and baseline characteristics

Demographic data will include, but are not limited to, sex, age, marital status, ethnicity, and family history. Baseline characteristics will include, but are not limited to, respiratory rate, heart rate, pulse, laboratory parameters, and relevant questionnaire scores (such as GCS and APACHE II). Comparisons between the EA and SEA groups will be performed to assess baseline comparability and confirm the adequacy of randomization.

4.2 Subgroup analyses

Pre-specified subgroup analyses will be conducted based on disease type (ischemic stroke, intracerebral hemorrhage, and subarachnoid hemorrhage), age groups, and sex. To assess potential interactions between treatment and subgroup factors, statistical models such as linear mixed-effects

models, generalized linear mixed models, or generalized estimating equations will be employed, with interaction terms included in the models.

4.3 Sensitivity analyses

Sensitivity analyses will be performed to assess the robustness of the primary outcome results, including separate analyses in the ITT and PP populations, and analyses excluding outliers or major protocol violators. Additional sensitivity analyses may be conducted using alternative statistical models or adjustment methods as appropriate.
